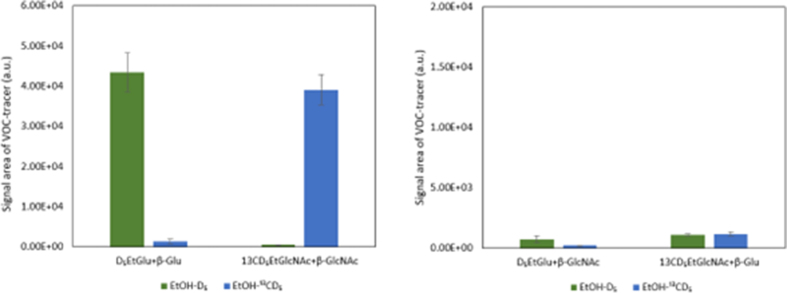# Induced volatolomics to uncover new enzymatic hallmarks of precancerous lesions: a proof of concept on gastric preneoplasia in mice

**DOI:** 10.1016/j.bbrep.2025.102062

**Published:** 2025-05-30

**Authors:** Rony Eid, Estelle Blochouse, Alban Giese, Sébastien Papot, Philippe Jay, Christine Varon, Pauline Poinot

**Affiliations:** a University of Poitiers, UMR CNRS 7285, Institut de Chimie des Milieux et Matériaux de Poitiers (IC2MP), Cedex 9, Poitiers, 86073, France; b University of Bordeaux, INSERM, UMR1312 BoRdeaux Institute of Oncology, F33000, Bordeaux, France; c University of Montpellier, CNRS UMR 5203, INSERM, Institute of Functional Genomics, Cedex 5, Montpellier, 34094, France

## Abstract

Induced volatolomics is an emerging field of research that offers new opportunities in biology by detecting volatile reporters released by activatable probes, enabling the exploration of oncogenic processes. Building on its proven efficiency in exploring the evolution of implanted tumours, we hypothesized that induced volatolomics could extend its application to detect precancerous conditions. As a proof of concept, we performed a longitudinal study and investigated glycosidase activity during the early stages of gastric carcinogenesis development induced by *Helicobacter felis* infection in mice, mimicking the gastric carcinogenesis cascade induced by chronic *Helicobacter pylori* infection in humans. We identified upregulated exoglycosidases linked to acute infections or inflammatory processes in tissues infected by Helicobacter. Specifically, α-mannosidase and β-galactosidase activities in stomach tissue were found to be strongly associated with the initial stages of *Helicobacter* infection. Additionally, the activities of β-Glucuronidase and β-N-acetyl-glucosaminidase increased during the progression to preneoplastic stages, potentially signalling the transition from infection to inflammation-driven carcinogenesis. These enzymes may serve as early biomarkers for detecting gastric carcinogenesis. Our study highlights the potential of VOC-based probes for real-time monitoring of gastric cancer progression through tissue biopsies. Therefore, this study demonstrates the potential of induced volatolomics for investigating biological processes and uncovering new therapeutic strategies.

## Highlights

• Induced volatolomics offers new opportunities by detecting volatile reporters released by activatable probes.
• We identified upregulated glycosidases linked to infection or inflammatory processes in tissues infected by Helicobacter.
• These enzymes may serve as early biomarkers for detecting gastric carcinogenesis.
• This study highlights potential of VOC-based probes for monitoring of gastric cancer progression through tissue biopsies.
• These findings could pave the way for more effective diagnostic strategies for early detection of carcinogenesis.

## 1 Introduction

Cancer is a multifactorial and highly complex group of diseases. Their initiation is often estimated to occur over several decades, during which defective molecular and cellular processes progressively accumulate. While significant therapeutic advancements have been made, it is likely that greater success could be achieved by intervening at the earliest stages of tumour development. This requires a thorough understanding of the biology of pre-tumoral states and the identification of markers that influence progression to malignancy. To address this, it is essential to develop and enhance multimodal approaches that enable precise, multidimensional characterization and classification of precancerous lesions based on their estimated risk of malignant transformation [1].

Induced volatolomics is an emerging field of research that offers new opportunities in biology by detecting volatile reporters released by activatable probes, enabling the exploration of oncogenic processes [2]. We demonstrated that probes based on volatile organic compounds (VOCs) can be activated *in vivo* by enzymes upregulated in solid tumours, releasing exogenous volatile tracers detectable in breath. This method allowed us to detect *in vivo* solid tumours, monitor their progression during chemotherapy, and aid in developing new enzyme-targeted drug candidates [[3], [4], [5]]. Considering its efficiency in exploring implanted tumours progression, we hypothesized here that induced volatolomics could go further and highlight precancerous condition. To answer this question, we further expanded our pool of VOC-based probes by up to seven probes, each targeting a specific glycosidase activity.

Thus, in this proof-of-concept study, we proposed a set of enzyme-sensitive probes for the multiplexing of glycosidase activities during the early stages of gastric carcinogenesis development. Indeed, despite advances in treatment, gastric cancer still has a very poor prognosis. With the death rate from gastric cancer projected to increase by 66 % by 2040 [6], focusing on prevention, early diagnosis, and new drug discoveries is essential. Since around 90 % of gastric cancer cases can be attributed to chronic infection with ***Helicobacter pylori*** [7], we decided to perform a longitudinal study to assess the early stages of gastric carcinogenesis development induced by *Helicobacter felis* infection in mice, mimicking the gastric carcinogenesis cascade induced by chronic *Helicobacter pylori* infection in humans (Fig. 1a).

**Fig. 1:**
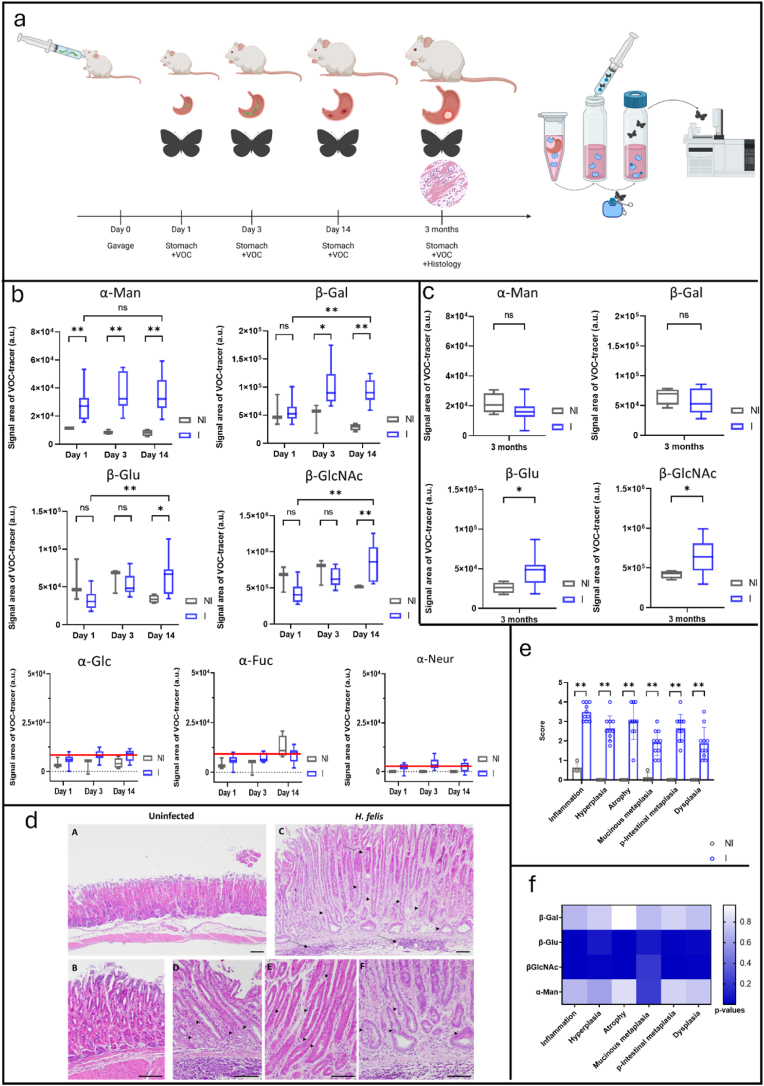
**a.** A volatolomics-based approach was developed to measure the dynamics of functional markers in gastric biopsies from 1 day to 3 months after Helicobacter infection. To highlight the dysregulation of glycosidase activity in stomach tissue sections, wild-type mice were inoculated via oral gavage with a suspension of *H. felis*. Control groups of non-infected mice received PBS alone. Mice were euthanized at 1 day, 3 days, 14 days, or 3 months post-infection. Stomachs were removed and sectioned longitudinally into pieces. Tissue sections were incubated for 30 min in DMEM medium, then removed, and the supernatants were centrifuged. VOC-based probes were then incubated in the supernatants for 4 h. Ethanol tracers were trapped for 30 min in the sample headspace and analysed by GC-MRMMS as previously described. Histology and immunohistochemistry experiments were performed on stomach sections from 3-month post-infection mice following the previously described protocol. **b.** Chemical evidence of α-Man, β-Gal, β-Glu, and β-GlcNAc activities in tissue samples from non-infected (NI) and infected (I) C57BL/6J female mice (n = 3 and n = 10 at day 1; n = 3 and n = 10 at day 3; n = 4 and n = 10 at day 14). test. α-Glc, α-Fuc, and α-Neur activities were not detected (ethanol signals were below their quantification limits, red bar, Fig. S1). **c.** α-Man, β-Gal, β-Glu, and β-GlcNAc activities in tissue samples from non-infected (NI) and infected (I) C57BL/6J male mice 3 months post infection (n = 4 and n = 10). **d.** Disease progression after 3 months *H*. *felis* infection in C57BL6 mice. Representative histopathologic features of noninfected (*A, B*) or *H. felis*-infected (*C, D, E, F*) stomachs are shown and pointed by the black arrows. A, B: No inflammation, no hyperplasia, no metaplasia. C: strong multifocal inflammation of the gastric mucosa invading the deeper layers (long arrows), hyperplasia with increase in mucosal height, metaplasia (arrow heads) and mild dysplasia. D: mucinous metaplasia (arrow heads) with cells resembling Brunner's glands. E: pseudo-intestinal metaplasia with enterocyte-like cells (arrow heads). F: mild dysplasia with cellular atypia and irregular shaped glands (arrow heads). A, C magnification x100. B, D, E, F magnification x200. Scale bars = 100 μm. **e**. Scoring of gastric histopathological criteria in mice. Gastric inflammation, hyperplasia, atrophy, mucinous metaplasia, pseudointestinal metaplasia, and dysplasia determined at 3-month post-infection. Data represent mean of scores ± SD for control noninfected (n = 4) and *H*. *felis*−infected mice (n = 10). **f.** Spearman's correlation test relating α-Man, β-Gal, β-Glu, and β-GlcNAc activities in 3-month post-infection mice and gastric histological scores. *p-value* scale is represented by the vertical blue bar. Kruskal-Wallis with post hoc Mann-Whitney or post hoc Dunn applied in b, c, and e. Mann-Whitney post-hoc test infected vs. uninfected control, ∗p < 0.05, ∗∗p < 0.01; Dunn post-hoc test, infected 24h vs infected 2 weeks ∗p < 0.05, ∗∗p < 0.01.

## 2 Results and discussion

We monitored the activity of extracellular glycosidases in gastric tissue samples after **bacterial** infection, as dysregulation of these enzymes could indicate infection, inflammation or cancer [[8], [9], [10], [11]]. We used seven labelled VOC-based probes, ethyl-β-d-galactopyranoside (EtGal), ethyl-α-d-glucopyranoside (EtGlc), ethyl-β-D-glucuronide (EtGlu), ethyl-α-l-fucopyranoside (EtFuc), ethyl-α-D-*N*-acetylneuraminic acid (EtNeur), ethyl-α-mannopyranoside (EtMan) and ethyl-*N*-acetyl-β-d-glucosamine (EtGlcNAc), targeting respectively β-galactosidase (β-Gal), α-glucosidase (α-Glc), β-glucuronidase (β-Glu), α-fucosidase (α-Fuc), α-D-*N*-acetyl-neuraminidase (α-Neur), α-mannosidase (α-Man) and β-*N*-acetyl-glucosaminidase (β-GlcNAc) and added them to native tissue sample supernatants. When activated by the corresponding enzyme, these probes released ethanol molecules labelled with either non-radioactive ^13^C or ^2^D or both [4]. Since tissues were not lysed, these different isotopes would thus be detectable as specific tracers of extracellular enzyme activities.

We first investigated glycosidase activities in gastric tissue samples of C57BL/6J mice at 1, 3 and 14 days after infection with *Helicobacter felis* (strain CS1), a *Helicobacter* species better adapted to mouse stomach colonization than *H. pylori* and which induces similar lesions as carcinogenic strains of *H*. *pylori* [12]. Tissue samples from non-infected animals were used as negative controls. The volatile tracers used to monitor the activation of the EtGlc, EtFuc, and EtNeur probes were within the limit of quantification (Fig. 1b, Fig. S1). These results suggest that all three glycosidases, α-Glc, α-Fuc, and α-Neur, were detected in the gastric mucosa either under normal conditions or after H. felis infection. However, these activities could not be quantified with sufficient reliability to allow a reliable comparison between uninfected and infected conditions. In contrast, we detected α-Man, β-Gal, β-Glu, and β-GlcNAc activities in tissue supernatants, indicating active secretion into the extracellular space. Compared to control mice, α-Man and β-Gal activities in stomach tissue doubled within a few days post-infection (specifically on days 1 and 3, respectively), reaching values 2.2 to 7.4 times higher for α-Man, and 2 to 4.3 times higher for β-Gal 14 days later. (Fig. 1b). β-Glu and β-GlcNAc also exhibited 1.8 and 1.5 fold increased catalytic activities, respectively, in gastric tissues two weeks after infection as compared to non-infected tissues (Fig. 1b).

Although the overexpression of glycosidases in bodily fluids and tissues has been reported in viral infection and chronic inflammation processes [4,5], the presence of elevated concentrations of α-Man, β-Gal, β-Glu, and β-GlcNAc in the extracellular environment of gastric tissues after *Helicobacter* infection had not been documented so far. Even though we did not observe a linear correlation between glycosidase activities and bacterial load immediately after infection, the early detection of α-Man and β-Gal activity may reflect the activation of glycosylation machinery required by the bacteria for pathogenesis or tissue remodeling in response to the infection. Therefore, these enzymes could serve as markers of acute infection. In contrast, the increase in β-Glu and β-GlcNAc activity in infected tissues 14 days later suggests a shift in pathological events. This may indicate a transition from infection to inflammation processes. Hence, we decided to investigate whether dysregulation of extracellular glycosidases could be a hallmark of chronic inflammation and premalignancy induced by *Helicobacter* infection. Thus, we applied our VOC-based probe strategy to stomach tissue sections 3 months post-infection. We also assessed gastric histopathological criteria as reported earlier [12,13] (Fig. 1a).

Interestingly, we observed a decrease in α-Man and β-Gal activities that were no longer upregulated in infected mouse tissues at that time (Fig. 1c). Such data tend to confirm their role in the primary infection process. These outcomes emphasize the potential of induced-volatolomics modalities to identify novel markers for *Helicobacter* infection.

In contrast, β-Glu and β-GlcNAc activities remained significantly higher in *Helicobacter*-infected mice tissues compared to the non-infected controls (Fig. 1c). Histopathological analysis confirmed that *H*. *felis* induced chronic inflammation, hyperplasia, mucosal atrophy, metaplasia, and dysplasia in these infected tissues (Fig. 1d and e). As shown earlier [12], these histological features, 15 weeks post infection, constitute hallmarks of gastric preneoplasia, that then evolve to cancerous lesions 20 weeks later. With the aim to explore possible correlations between induced volatolomics and histological data, we performed Spearman correlation test (Fig. 1f). Remarkably, the upregulation of extracellular β-Glu and β-GlcNAc activities strongly correlated with various levels of inflammation. Specifically, these enzymes could be used as markers of chronic inflammation, atrophy, and pseudointestinal metaplasia and dysplasia (0.002 < p-values <0.014). Although the overexpression of β-Glu and β-GlcNAc in well-established tumours has already been reported [5], their involvement in the development of precancerous lesions had not been documented before. Since their activities remained high and constant from 2 weeks to 3 months post-infection, these enzymes could serve as early markers of gastric carcinogenesis. Under these circumstances, the longitudinal use of our VOC-based probes on gastric tissue biopsies should be highly valuable for real-time monitoring of disease progression. Hence, our induced volatolomics strategy could represent a smart alternative for the early detection of patients at high risk of gastric cancer. Indeed, recent studies have highlighted that there are currently no excellent tumour markers for gastric cancer. Carcinoembryonic antigen (CEA) and carbohydrate antigen 19–9 (CA19-9) are commonly used tumour markers. These markers correlate with disease progression, including depth of invasion, lymph node metastasis and TNM stage [14]. However, they have limited diagnostic value for early stages of gastric cancer, they are useful for detecting advanced disease and monitoring treatment efficacy [15]. Similarly, immunohistochemical markers, including MLH1, p53, HER2, and E-cadherin, have been proposed as candidates for gastric cancer classifications, but their use is still limited by inconsistent results and difficulties in interpretation [16]. On the other hand, by monitoring the activities of these glycosidases in serum of patients infected by Helicobacter, we may be able to indicate steady increase in β-Glu and β-GlcNAc activities that would reflect a transition from infection to chronic inflammation, and preneoplastic changes. Within this framework, VOC-based probes could provide crucial information on changes in enzymatic activity associated with the progression of bacteria based-cancer. In this context, our strategy opens a new way for investigating oncomicrobes such as Fusobacterium which promotes colorectal or pancreatic cancers [17]. Indeed, the design of probes specific to oncobacterial enzymes would make it possible to highlight bacterial microniches within the tumor. In addition, the impact of the secretome of infected cancer cells on the remodeling of the extracellular matrix [18] could be studied.

Our study does, however, have certain limitations, including a small sample size and a relatively short follow-up period after infection. Indeed, longer monitoring of the animals would have provided us with information on the evolution of preneoplastic lesions towards neoplasia, thus confirming the robustness of β-Glu and β-GlcNAc as indicators of preneoplasia.

Additionally, this preliminary work should be deepened with data explaining the release of exoglycosidases into the tissue microenvironment during the onset of inflammation. Investigating these mechanisms through cell culture models could help generate hypotheses and provide deeper insights into these processes.

On the other hand, the study's strengths lie in the high selectivity and sensitivity of VOC-based probes. By employing multiple isotopes of the same volatile tracer (e.g., ethanol), we developed VOC-based probe cocktails capable of discriminating between multiple catalytic events in real time, even within a complex biological matrix. This represents a significant advantage over fluorescent probes, which cannot be used in cocktail form due to imaging interferences. Furthermore, our strategy demonstrates major advancements in sensitivity and normalization, as the biological matrix does not interfere with the detection of exogenous volatile tracers.

In conclusion, this proof on concept study demonstrated that the induced-volatolomics is an emerging area of research that offers opportunities to uncover new biochemical pathways involving exoglycosidases during the early stages of gastric cancer development. Identifying these preneoplasia-associated enzymes could expand drug target space and accelerate the development of novel therapeutic strategies in the future.

## CRediT authorship contribution statement

**Rony Eid:** Methodology, Investigation, Formal analysis. **Estelle Blochouse:** Methodology, Investigation, Formal analysis. **Alban Giese:** Methodology, Investigation, Formal analysis. **Sébastien Papot:** Validation, Project administration. **Philippe Jay:** Validation, Resources, Project administration. **Christine Varon:** Writing – review & editing, Validation, Supervision. **Pauline Poinot:** Writing – review & editing, Writing – original draft, Supervision, Conceptualization.

## Data availability

All data supporting the findings of this study are available within the main text and supplemental material and from the corresponding authors upon request**.**

## Ethics approval

Animal experiments on C57BL6J wild type mice were performed at the level 2 animal facilities of Bordeaux University (agreement number B33063916) with the approval of the Animal Experimentation Ethics Committee CEEA50 in conformity with the French Ministry of Agriculture Guidelines on Animal Care (approval numbers A38002 and A34219).

## Funding information

The authors thank the local committees Vienne and Deux-Sèvres of the Cancer League and the National Cancer League, and the “Région Nouvelle Aquitaine” (AAPR2022-2021-17103510 and AAPR2022-2021-17316710) for financial supports. This work has also been funded by the ANR through the Young Researcher grant “Volatolomix” (ANR-21-CE18-0009). This work was supported by the Canceropole Grand Sud-Ouest (Grant Emergence). We also thank the CNRS which supplied the project through the “AAP Emergence”. We thank the National Reference Centre for Helicobacters and Campylobacters (University Hospital of Bordeaux) for technical assistance with Helicobacter culture.

## Declaration of competing interest

The authors declare the following financial interests/personal relationships which may be considered as potential competing interests: POINOT PAULINE reports financial support and equipment, drugs, or supplies were provided by National League Against Cancer. Poinot Pauline reports financial support and equipment, drugs, or supplies were provided by Nouvelle-Aquitaine Regional Council. Varon Christine reports financial support and equipment, drugs, or supplies were provided by Nouvelle-Aquitaine Regional Council. Poinot Pauline reports financial support, article publishing charges, and equipment, drugs, or supplies were provided by French National Research Agency. If there are other authors, they declare that they have no known competing financial interests or personal relationships that could have appeared to influence the work reported in this paper.

## Figures and Tables

**figs1:**
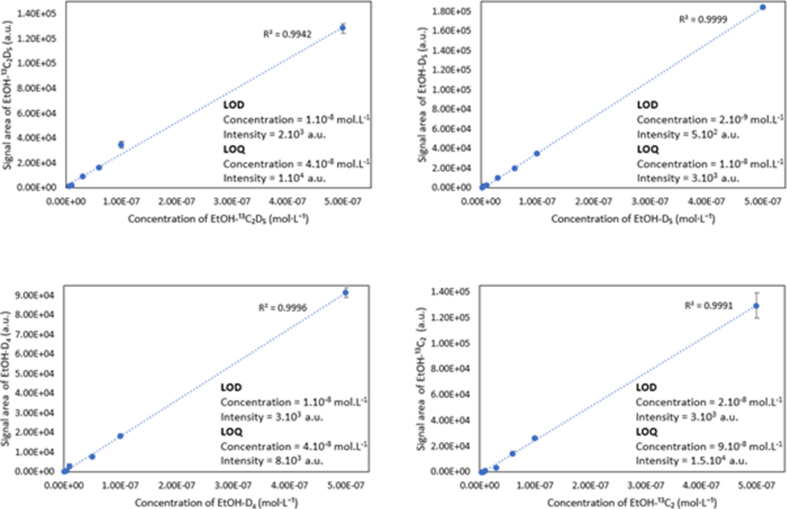


**figs2:**