# Supplementary material for: Induced volatolomics to uncover new enzymatic hallmarks of precancerous lesions: a proof of concept on gastric preneoplasia in mice
Source: Biochem Biophys Rep. 2025 May 30;43:102062. doi: 10.1016/j.bbrep.2025.102062 (PMC12166735; doi:10.1016/j.bbrep.2025.102062)
Supplement: Multimedia component 1 [file mmc1.docx]

**Supporting Information**

**Mouse handling**

Animal experiments on C57BL6J wild type mice were performed at the level 2 animal facilities of Bordeaux University (agreement number B33063916) with the approval of the Animal Experimentation Ethics Committee CEEA50 in conformity with the French Ministry of Agriculture Guidelines on Animal Care (approval numbers A38002 and A34219). Five-week-old male or female C57BL/6J mice free of pathogenic viruses, parasites and bacteria including *Helicobacter* species were purchased from Charles River Laboratories (L’Arbresle, France). Mice were housed five per microisolator cage on ventilated shelves on a 12-hour day/night cycle with constant humidity and temperature control, with low content vitamin diet and water *ad libidum*. Mice were euthanized prior to experimental endpoint if weight loss exceeded 20% of the starting weight.

***H. felis* infection**

*H. felis* strain ATCC 49179 was grown on Columbia agar plates under microaerobic conditions (N2: 85%; O2: 5%; CO2: 10%) at 37°C as previously described [12]. For mouse infection (n=40), bacteria were collected from agar plates and suspended in PBS and then inoculated into pre-fasted 6-7-weeks old mice by oral gavage for 3 consecutive days as previously described [12]. Uninfected control groups (n=14) were given PBS alone. Larger groups of infected mice were done to compensate for possible death of infected animals along the study. Mice were housed 5 per cage and they all benefited from the same housing conditions. Successful infection in the *H. felis*-infected group as well as the absence of infection in the control group were confirmed by quantitative real-time PCR experiments on the *H. felis* *fla*A gene performed on DNA extracted from fresh gastric tissue sample collected aseptically from each mouse as previously described[12,13].

**Tissue processing for histopathological and molecular analyses**

After mice euthanasia by cervical dislocation, stomachs were collected aseptically, opened along the greater curvature then washed in PBS before tissue sampling. For histological analysis, a part representing half of the stomach was divided into 2 longitudinal strips along the smaller curvature and from the squamocolumnar junction through the pylorus, fixed for 24 h in 3.7 % neutral-buffered formalin solution (Sigma Aldrich, Saint louis, USA), followed by standard histological procedure and paraffin embedding. Additional fresh longitudinal tissue strips representing approximately 1/8th of the glandular stomach (excluding the region of the stomach composed of stratified epithelium) were immediately collected into sterile tubes for molecular analysis (*fla*A RTqPCR) and snap-frozen on dry ice and stored at -80 °C prior to induced volatolomics analysis.

**Histology and histological scoring**

Tissue sections of 3 µm thickness were prepared from the formalin-fixed paraffin-embedded (FFPE) tissues and processed for Hematoxylin/Eosin (HE) staining as previously described [12]. Relative double-blinded scoring of lesions of the gastric mucosa was carried out on HE-stained tissue sections using a numerical scale of 1- 4 for quantification of inflammation, hyperplasia (mucosal height), oxyntic atrophy, mucinous metaplasia, pseudo-intestinal metaplasia and dysplasia as described previously [12]. Quantification values represent the mean of scores determined ± standard deviation (SD) in each group of mice. Representative images of the lesions observed were recorded using a NIKON Eclipse Ci phase contrast microscope equipped with the NIKON DSRi2 camera and NIS-BR software (NIKON, Champigny-sur-Marne, France) using 10x and 20x objectives.

**Induced volatolomics modality**

D_2_-ethyl-β-D-galactoside (D_2_-EtGal), ^13^C_2_-ethyl-β-D-galactoside (^13^C_2_-EtGal), D_4_-ethyl-α-D-fucoside (D_4_-EtFuc), D_5_-Ethyl-β-D-glucuronide (D_5_-EtGlu), ^13^CD_5_-N-acetyl-β-D-glucosamide (^13^CD_5_-EtGlcNac), ^13^C_2_D_5_-N-acetyl-β-D-glucosamide (^13^C_2_D_5_-EtGlcNac), D_4_-Ethyl-α-D-glucopyranoside (D_4_-EtGlc), D_5_-Ethyl-α-D-mannopyranoside (D_5_-EtMan), ^13^C_2_D_5_-Ethyl-α-D-mannopyranoside (^13^C_2_D_5_-EtMan), and **D_5_-ethyl-α-D-N-acetylneuraminic acid (D_5_-EtNeur)** were synthesized by @rtMolecule (Poitiers, France). Standards of ethanol (D_2_-ethanol, D_5_-ethanol, ^13^CD_5_-ethanol, ^13^C_2_D_5_-ethanol, and ^13^C_2_-ethanol) were purchased from Sigma-Aldrich (Saint louis, USA), and D_4_-ethanol was purchased from Cil Cluzeau info labo (Quebec, Canada).

**VOCs Preconcentration and analysis**

VOCs were trapped on a 75 μm carboxen/polydimethylsiloxane (CAR/PDMS) SPME fibre Sigma-Aldrich (Saint louis, USA). They were analysed with a GC-MS/MS system that consisted of a Trace 1300 Thermo Scientific gas chromatograph coupled with a TSQ 9000 triple quadrupole mass spectrometer (Thermo Fisher Scientific, Waltham, MA, USA) operated in multiple reaction monitoring (MRM MS).

After SPME fibre desorption, VOCs were separated on a non-polar capillary column DB-624 60 m x 250 µm x 1,4 μm (Agilent, Santa Clara, USA). The injector temperature was set at 260°C in splitless mode. Septum purge flow and gas saver flow were set to 5 mL.min^-1^ and 20 mL.min^‑1^ respectively. SPME fibre desorption was performed with helium as the carrier gas with a 1.2 mL.min^-1^ flow. An isotherm program was used to separate VOCs on the column. Oven temperature was set at 90 °C during 5 min.

Multiple reaction monitoring (MRM) transitions and collision energies for ethanol isotopes were as follows: for D_2_-ethanol, 48.1 > 33.1 with 5 eV; for D_4_-ethanol, 50.1 > 32.1 with 5 eV; for D_5_-ethanol, 51.1 > 33.1 with 5 eV; for ^13^CD_5_-ethanol, 52.1 > 34.1 with 6 eV; for ^13^C_2_D_5_-ethanol, 53.1 > 34.1 with 5 eV and for ^13^C_2_-ethanol, 48.1 > 32.1 with 5 eV.

Every day, trapping efficiency of SPME fibre was verified using a 300 µL solution at 10^-7^ mol.L^‑1^ of D_2_-ethanol, D_4_-ethanol, D_5_-ethanol, ^13^CD_5_-ethanol, ^13^C_2_D_5_-ethanol and ^13^C_2_-ethanol in ultra-pure water. Headspace-SPME trapping was performed during 30 min at 37 °C. Ethanol isotopes were analysed with the GC-MRM MS method described above.

To assess the sensitivity of this GC-MRM MS method, a five-points calibration curve ranging from 6.10^-9^ to 5.10^-7^ mol.L^-1^ was constructed for four ethanol isotopes (^13^C_2_, D_4_, D_5_, ^13^C_2_D_5_) (two replicates per calibration point). Ethanol molecules were trapped during 30 min with a SPME fibre after 2 h 00 incubation at 37°C. The amount of labelled-ethanol trapped on the SPME fibre was analysed with the GC-MRM MS method (Fig. S1). Limit of detection and quantification were determined for the four molecules.

*Fig. S1. Calibration curves for ^13^C_2_D_5_-ethanol, D_5_-ethanol, D_4_-ethanol and^13^C_2_-ethanol. Limit of detection and quantification are specified on each graph.*

**Enzyme activities in stomach tissues**

Since the probes will be used in cocktails to simultaneously demonstrate multiple glycosidase activities in tissues, we first investigated potential cross-reactivities between glycosidases. For proof of concept, we incubated β-GlcNAc with the EtGlu probe and, conversely, β-Glu with the EtGlcNAc probe (Fig. S2). These tests demonstrated that β-GlcNAc does not convert the EtGlu probe to D_5_-ethanol, and similarly, β-Glu had no effect on the EtGlcNAc probe. In contrast, in the presence of their corresponding probes, both enzymes converted probe substrates to ethanol isotopes. These assays illustrated the high specificity of the glycosidases for their probe substrate.

*Fig. S2. Study of cross-reactivities between enzymes. Incubation of β-GlcNAc enzyme with either its probe substrate (i.e. EtGlcNAc probe) or β-Glu substrate (i.e. EtGlu). The same experiment was performed with β-Glu enzyme.*

Tissue samples from non-infected (NI) and infected (I) C57BL/6J female mice (n=3 and n=10 at day 1; n=3 and n=10 at day 3; n=4 and n=10 at day 14) and C57BL/6J male mice 3 months post-infection (n=4 and n=10) were weighed and placed in 2 mL tubes. A volume of 1 mL of DMEM high glucose, without glutamine (11960085, Modified Eagle Medium; Thermo Fisher Scientific, Illkirch, France) was added and incubated for 30 minutes at 37 °C. The tissues were then removed, and the medium was centrifuged (1000 rpm, 4 °C, 15 min). A 75 µL aliquot of the supernatant was placed in a 3 mL clear glass vial with 1X (11 µL) protease inhibitor (11836170001, Roche Diagnostics GmbH, Mannheim, Germany), 184 µL of acetate buffer (0.01 M, pH 5), and 30 µL of probes (probe final concentration of 5.10⁻⁴ M for D_5_-EtNeur and 10^-3^ M for all others). The mixture was incubated for 4 hours at 37 °C. The vials were hermetically sealed with a Mininert® valve screw cap (Sigma Aldrich, Saint louis, USA). After 4 hours of reaction, an SPME fibre was inserted into the headspace and exposed to trap ethanol isotopes for 30 minutes. Trapped VOCs were analysed by GC-MRM MS as described above. Two vials were prepared per tissue sample.

In parallel, a set of blank samples were prepared. Some blanks consisted of 30 µL of probes, 11 µL of protease inhibitor, 184 µL of acetate buffer, and 75 µL of DMEM. Others consisted of 75 µL of the centrifuged medium (after tissue removal) ,11 µL of protease inhibitor and 214 µL of acetate buffer. These were processed by exposing an SPME fibre for 30 minutes in the sample headspace. Trapped VOCs were analysed using the GC-MRM MS method as described above. The blank experiments revealed very low contamination corresponding to the spontaneous hydrolysis of a few probes. Consequently, all ethanol isotope signals were corrected by subtracting the background noise.

For data treatment, all ethanol isotope signals were standardized by the weight of tissue sample and the SPME fibre daily trapping efficiency.

Statistical analysis

Data are mean ± s.e.m. All statistical treatments were performed using XLStat 4 (Paris, France). Significant differences were determined by Mann-Whitney test. A p-value < 0.05 was considered statistically significant. Spearman’s correlation test was performed to determine the correlation between enzyme activities and gastric histological scores. Any missing data was rejected.
